# Prognostic relevance of MIB-1 labeling index in VHL-associated and sporadic spinal hemangioblastomas: a subgroup analysis from a multicentric study

**DOI:** 10.1186/s40478-025-02202-w

**Published:** 2025-12-11

**Authors:** Johannes Wach, Alim Emre Basaran, Obada T. Alhalabi, Jürgen Beck, Vicki M. Butenschoen, Steven D. Chang, Marcus Czabanka, Tomasz Czernicki, Philipp Dammann, Roberto Doria-Medina, Sven Oliver Eicker, Alonso Barrantes-Freer, Christine Gizaw, Erdem Güresir, Marc Hohenhaus, Romina Hohenhaus, Ahed H. Kattaa, Fatma Kilinc, Lukas Klein, Nikolaus Kögl, Sandro Krieg, Przemyslaw Kunert, Maximilian Middelkamp, Bernhard Meyer, Nicolas Neidert, Julia Onken, Tobias Pantel, David J. Park, Laurèl Rauschenbach, Roman Sankowski, Alejandro N. Santos, Nils Ole Schmidt, Sebastian Siller, Ulrich Sure, Claudius Thomé, Tarik Tihan, Martin Vychopen, Peter Vajkoczy, Maria Wostrack, Jan-Helge Klingler

**Affiliations:** 1 Department of Neurosurgery, University Hospital Leipzig, Liebigstrasse 20, 04103 Leipzig, Germany; 2 Comprehensive Cancer Center Central Germany, Partner Site Leipzig, Leipzig, Germany; 3 Division of Neuropathology, UCSF School of Medicine, San Francisco, CA USA; 4 Department of Pathology, Miller School of Medicine, University of Miami, Florida, USA; 5 Department of Neurosurgery, School of Medicine, Technical University of Munich, Klinikum rechts der Isar, Munich, Germany; 6 Department of Neurosurgery, University Hospital, University of Regensburg, Regensburg, Germany; 7 Department of Neurosurgery, Charité—Universitätsmedizin Berlin, Corporate Member of Freie Universität Berlin and Humboldt-Universität zu Berlin, Berlin, Germany; 8 Department of Neurosurgery and Spine Surgery, University Hospital Essen, Essen, Germany; 9 Department of Neurological Surgery, University of Miami Miller School of Medicine, Miami, FL USA; 10 DKFZ-Division Translational Neurooncology at the WTZ, DKTK Partner Site, University Hospital Essen, Essen, Germany; 11 Department of Neurosurgery, Medical Center—University of Freiburg, Freiburg im Breisgau, Germany; 12 Department of Neurosurgery, Heidelberg University Hospital, Heidelberg, Germany; 13 Medical Faculty, Heidelberg University, Heidelberg, Germany; 14 Department of Neurosurgery, Medical University Innsbruck, Innsbruck, Austria; 15 Department of Neurosurgery, Medical University of Warsaw, Warsaw, Poland; 16 Department of Neurosurgery, University Medical Center Hamburg-Eppendorf, Hamburg, Germany; 17 Department of Spine and Scoliosis Surgery, Lubinus Clinicum, Kiel, Germany; 18 Department of Neurosurgery, Stanford University School of Medicine, Stanford, CA USA; 19 Department of Neurosurgery, University Hospital Frankfurt, Frankfurt am Main, Germany; 20 Paul-Flechsig-Instiute of Neuropathology, University of Leipzig Medical Center, 04103 Leipzig, Germany; 21 Institute of Neuropathology, Faculty of Medicine, Medical Center—University of Freiburg, 79106 Freiburg, Germany; 22 Department of Neurosurgery, Klinikum Main-Spessart, Lohr am Main, Germany

**Keywords:** Spinal hemangioblastoma, Von Hippel-Lindau disease, Local tumor progression, MIB-1 index, Progression-free survival

## Abstract

Spinal hemangioblastomas (sHB) are rare vascular tumors, with distinct clinical courses between von Hippel-Lindau (VHL)-associated and sporadic cases. The MIB-1 labeling index has been proposed as a surrogate marker for tumor proliferation, but its prognostic value remains unclear in this context. In this subgroup analysis from a multicenter retrospective study, we analyzed 116 primary sHB patients with available MIB-1 indices. Patients were stratified by VHL status. Statistical comparisons included ROC analyses for local progression-free survival (PFS) prediction and Kaplan–Meier survival curves for local PFS, stratified by a MIB-1 index cut-off derived from Youden’s index. The MIB-1 index was significantly lower in VHL-associated tumors compared to sporadic ones (mean 2.17% vs. 3.02%, *p* = 0.008). In VHL-associated sHB, a higher MIB-1 index (≥ 2%) correlated with an increased risk of local tumor progression (AUC 0.74, 95% CI 0.49–0.98), whereas this was not observed in sporadic cases (AUC 0.56, 95% CI 0.23–0.88). Kaplan–Meier analysis showed that VHL patients with MIB-1 ≥ 2% had significantly shorter PFS (*p* = 0.05), while no significant association was found in sporadic tumors (*p* = 0.87). Our findings suggest that while VHL-associated sHB exhibit lower proliferative indices overall, elevated MIB-1 labeling indices might serve as a prognostic marker of shorter local PFS in this subgroup. In contrast, MIB-1 index appears to have limited prognostic relevance in sporadic sHB. These results highlight the importance of further molecular stratification and proliferation assessment in sHB to better inform clinical decision-making.

**Graphical abstract:**

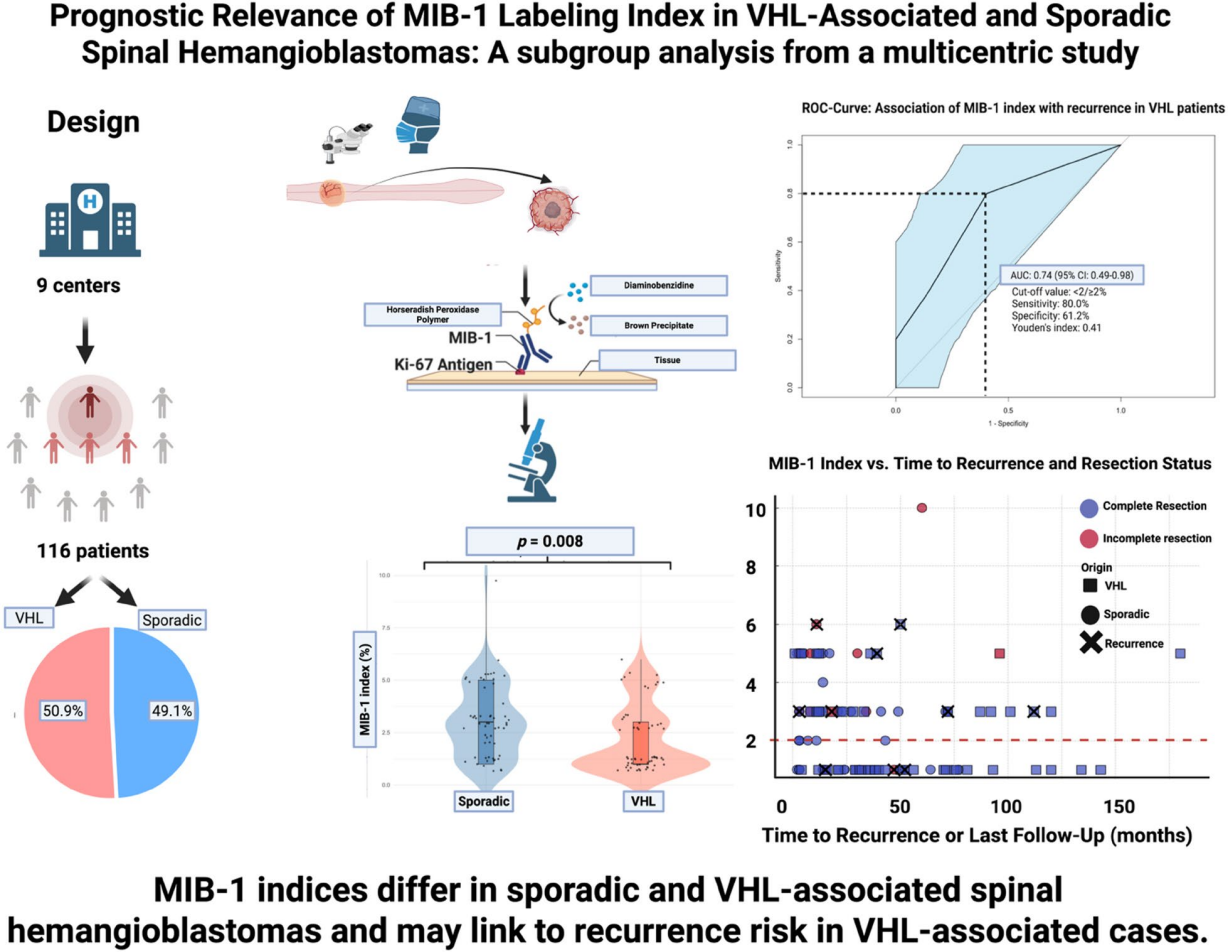

**Supplementary Information:**

The online version contains supplementary material available at 10.1186/s40478-025-02202-w.

## Introduction

Spinal hemangioblastomas (sHB) are rare, benign, and highly vascularized tumors of the central nervous system (CNS) and account for approximately 2–6% of all spinal neoplasms [1]. These tumors can occur either sporadically or in association with the autosomal dominant inherited von Hippel-Lindau (VHL) syndrome [2]. In patients with VHL-associated sHB, lesions are frequently multifocal and may recur or newly develop at various levels along the neuroaxis. Furthermore, VHL-associated cases tend to occur at a younger age and are associated with a higher risk of shorter local PFS compared to sporadic cases [3].

Despite the benign nature of sHB, intramedullary tumor growth can lead to significant neurological deficits and a deterioration in quality of life (QoL). Surgical resection remains the first-line therapy in the treatment of sHB, and in cases of complete resection, it is considered curative [4, 5]. However, local progression can still occur, especially in VHL-associated cases, highlighting the need for reliable predictive biomarkers to assess tumor behavior and guide clinical decision-making [6]. A potential biomarker for tumor proliferation is the MIB-1 labeling index, which quantifies the expression of the Ki-67 cell cycle protein through immunohistochemistry [7]. While the MIB-1 index has been established as a valid and reliable prognostic marker for tumor progression and survival in various CNS tumors, its prognostic value in sHB remains largely unexplored [8–10]. In particular, it is still unclear whether there are differences in MIB-1 expression between VHL-associated and sporadic sHB, and to what extent the MIB-1 index is associated with local PFS [11].

The aim of the present subgroup analysis was to investigate the role of the MIB-1 index in patients with both sporadic and VHL-associated sHB. This subgroup analysis is based on a multicenter retrospective cohort previously described by Wach et al. [12], where 357 patients with sHB were analyzed with respect to local tumor control and neurological outcomes after surgery. In the current study, we focus on a subset of these patients for whom MIB-1 labeling index data was available. In particular, special attention was given to the association between MIB-1 index and local PFS.

## Materials and methods

### Study design and inclusion criteria

**Fig. 1:**
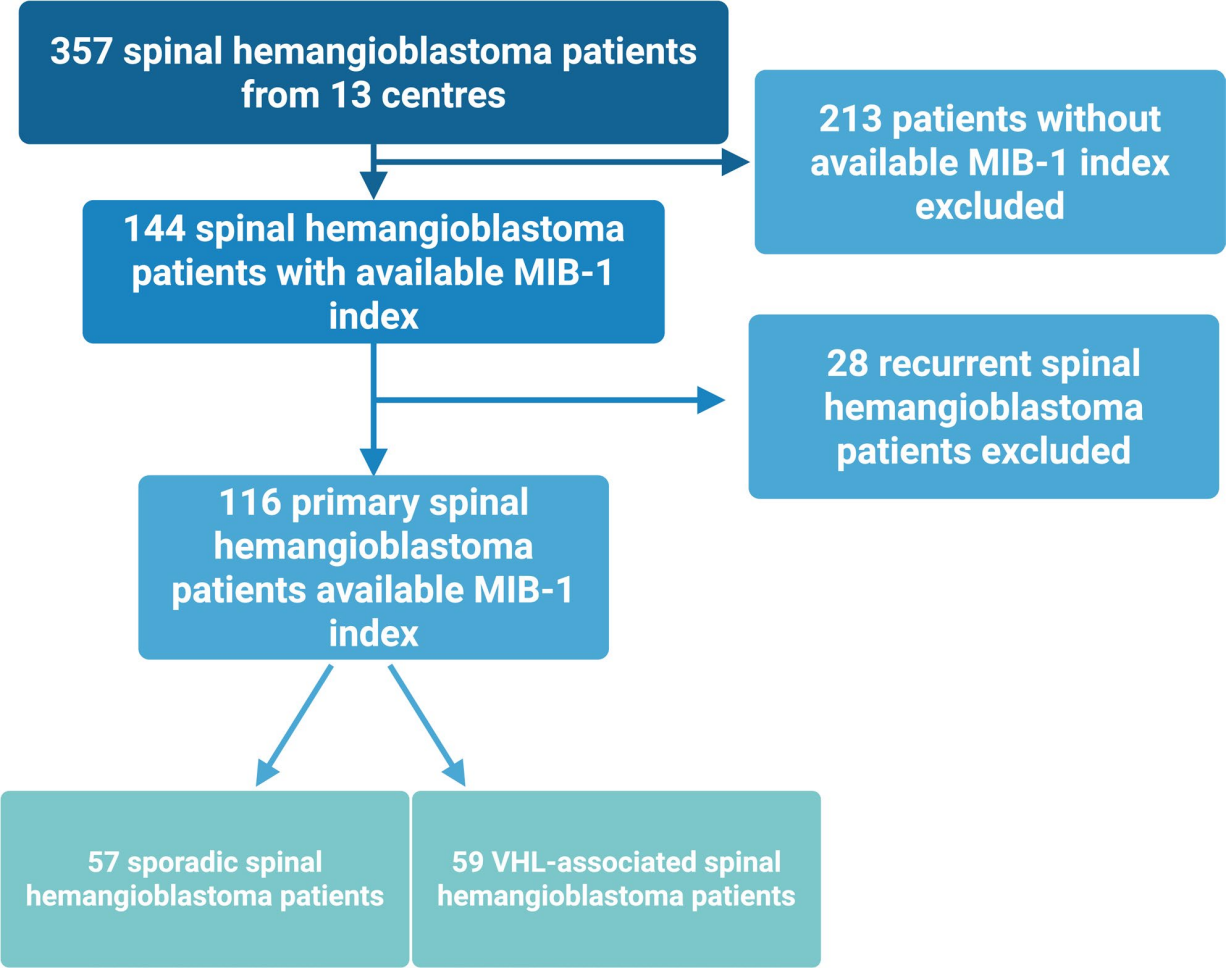
Flowchart of patient selection

We retrospectively identified 357 patients with histologically confirmed sHB from 13 participating centers. Of these, 213 patients were excluded for this subgroup analysis due to missing MIB-1 index data. Among the remaining 144 patients with an available MIB-1 index, 28 patients with recurrent sHB were excluded to ensure analysis of primary tumors only without any prior therapies. A final cohort of 116 patients from 9 centers with primary sHB and available MIB-1 index was included in this study. These patients were further stratified into two groups based on clinical and genetic background: 57 patients with sporadic and 59 patients with VHL-associated sHB. The workflow is summarized in Fig. 1.

### Data collection

The following clinical and radiological data were retrospectively extracted based on a defined study protocol as previously reported: age, sex, VHL status, spinal compartment involvement (intramedullary, extramedullary, combined) tumor localization (cervical, cervicothoracic, thoracic, thoracolumbar, lumbar, lumbosacral), number of involved spinal segments (solitary vs. multiple), presence of cysts and syrinx, preoperative hemorrhage, and extent of resection (EoR) (complete vs. incomplete). Local tumor progression was defined as either the recurrence of the tumor at the same site after complete resection (CR) or regrowth following partial resection [12]. Postoperative imaging follow-up was performed according to institutional standards across the 13 participating centers, resulting in heterogeneous surveillance intervals. In general, VHL-associated patients were monitored within dedicated VHL programs with regular craniospinal MRI approximately, whereas sporadic cases typically underwent MRI at the local tumor site at 3–6 months postoperatively and subsequently at 12–24-month intervals.

### MIB-1 labeling index assessment

To assess tumor proliferative activity, the MIB-1 (Ki-67) labeling index was determined in all available tumor specimens. The MIB-1 index was determined by immunohistochemical staining of formalin-fixed, paraffin-embedded tumor tissue. Immunohistochemical staining was performed using the monoclonal MIB-1 antibody (Dako, Denmark) according to standard protocols. Numerical MIB-1 indices were retrospectively extracted from final pathology reports. All MIB-1 labeling indices were determined manually according to local institutional protocols, with hotspot-based assessment used as the preferred counting strategy. Given the 15-year study period and involvement of multiple observers across 13 participating centers, staining and counting procedures were not fully standardized, reflecting routine clinical practice in a multicenter retrospective cohort.

### Statistical analysis

Patients were stratified into two groups based on VHL-associated and sporadic sHB. The MIB-1 index between these two groups was compared using two-sided independent t-test. The optimal cut-off for stratifying local PFS was determined using Youden’s index derived from receiver operating characteristic (ROC) analysis. The prognostic relevance of the MIB-1 index with respect to local PFS was assessed using Kaplan–Meier analysis and the log-rank test. Additionally, a multivariate binary logistic regression analysis was performed to identify independent predictors of MIB-1 labeling index. The analysis for predictors of increased MIB-1 labeling index in the entire cohort was performed using the median-split method with dichotomization cut-off set at the median value of 3% (≥ 3/ <), ensuring balanced group sizes and robust statistical comparison across both VHL-associated and sporadic sHB [13]. Furthermore, an optimal MIB-1 index cut-off regarding local PFS was calculated using Youden´s index from ROC analysis in both sporadic and VHL-associated sHB patients [14]. Progression-free survival analyses were conducted in the 97 of 116 patients for whom both MIB-1 labeling indices and follow-up data were available. Statistical analyses were conducted using SPSS software (version 29.0; IBM, Armonk, NY, USA). Kaplan–Meier survival curves were generated using R software (version 4.3.1) with the *survival* and *survminer* packages. A p-value of < 0.05 was considered statistically significant.

## Results

### Patient cohort and characteristics

A total of 116 patients with primary sHB were included in the analysis. The median age was 47.5 years (IQR: 31–57). Of the patients, 58 (50%) were female and 58 (50%) were male. In 59 patients (50.9%), a VHL syndrome was associated, whereas 57 patients (49.1%) had sporadic sHB.

In 70 patients (60.3%), the tumor localization was intramedullary, in 24 patients (20.7%), it was extramedullary and in 22 patients (19.0%) the tumor showed a combined localization. Regarding anatomical distribution, the cervical spine was affected in 51 patients (44.0%), cervicothoracic in 12 (10.3%), thoracic in 26 (22.4%), thoracolumbar in 14 (12.1%), lumbar in 10 (8.6%), and lumbosacral in 3 patients (2.6%).

In 73 patients (62.9%), only one spinal segment was involved, in 25 patients (21.6%) two segments, in 12 patients (10.3%) three segments and in six patients (5.2%) four segments were involved. Cysts were present in 47 patients (40.5%), and a syrinx was identified in 43 patients (37.1%). Preoperative bleeding was detected in seven patients (6.0%). Regarding the EoR, complete tumor removal was achieved in 101 patients (87.1%), while 15 patients (12.9%) underwent incomplete resection. For a detailed summary of patient characteristics, see Table 1.

**Table 1:** Patient characteristics

Variable	Total cohort (*n* = 116)
Age, median (IQR)	47.5 (31–57)
Female, n patients (%)	58 (50.0%)
VHL, n patients (%)	59 (50.9%)
Intramedullary, n patients (%) Extramedullary, n patients (%) Combined, n patients (%)	70 (60.3%) 24 (20.7%) 22 (19.0%)
Cervical, n patients (%) Cervicothoracic, n patients (%) Thoracic, n patients (%) Thoracolumbar, n patients (%) Lumbar, n patients (%) Lumbosacral, n patients (%)	51 (44.0%) 12 (10.3%) 26 (22.4%) 14 (12.1%) 10 (8.6%) 3 (2.6%)
Multiple lesions, n patients (%)	25 (21.6%)
*Number of involved segments, n patients (%)*	
1	73 (62.9%)
2	25 (21.6%)
3	12 (10.3%)
4	6 (5.2%)
*Cyst*	
Present, n patients (%)	47 (40.5%)
*Syrinx*	
Present, n patients (%)	43 (37.1%)
*Preoperative bleeding*	
Present, n patients (%)	7 (6.0%)
*Extent of resection, n patients (%)*	
Complete resection	101 (87.1%)
Incomplete resection	15 (12.9%)
MIB-1 index, median (IQR)	3.0 (1.0–10.0)

### MIB-1 labeling index

**Fig. 2:**
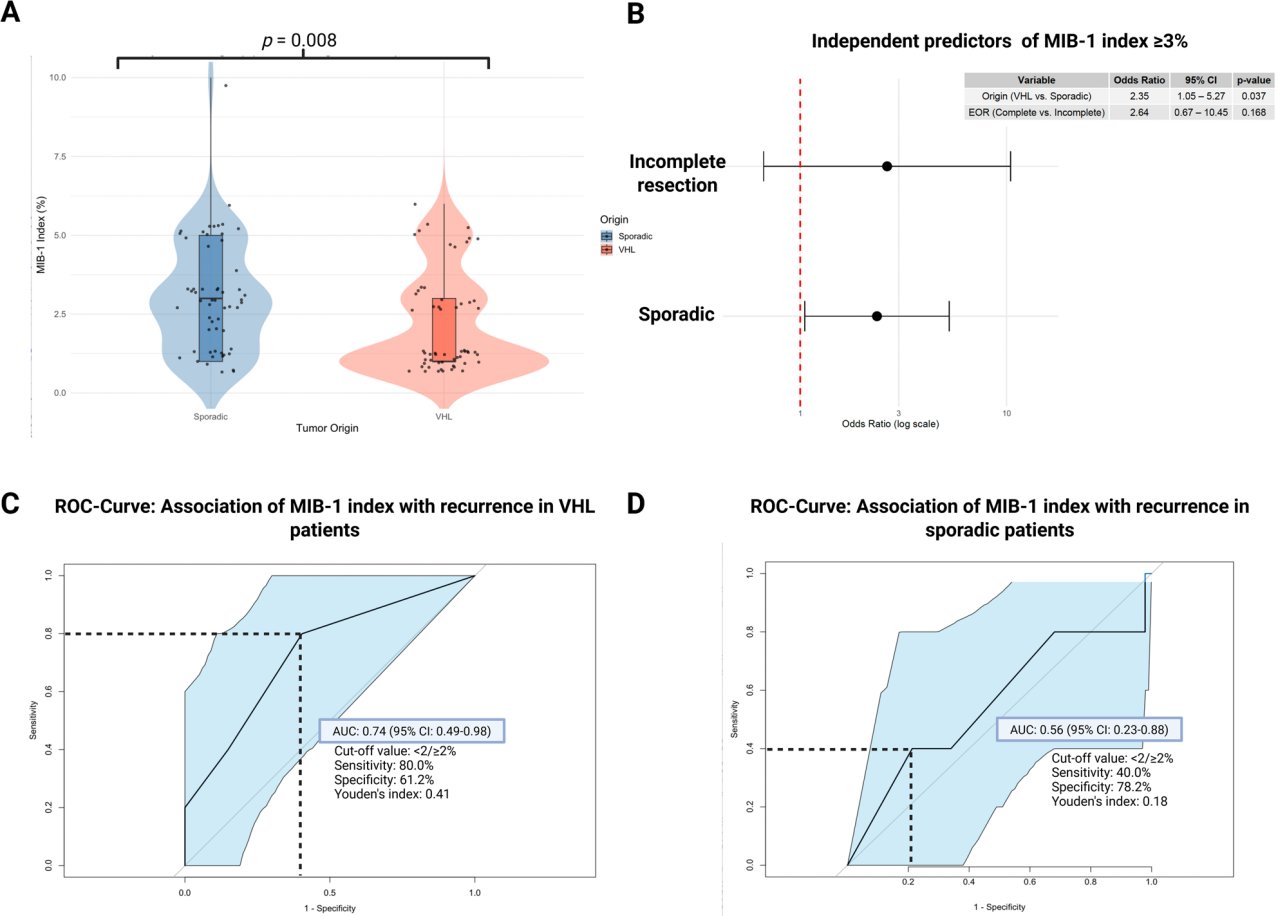
**A** Comparison of MIB-1 labeling index between sporadic and VHL-associated sHB. The MIB-1 index was significantly lower in VHL-associated tumors (*p* = 0.008). **B** Forest plot illustrating predictors of elevated MIB-1 index based on a cut-off value of ≥ 3% in the logistic regression model. Sporadic tumors had a higher OR for increased MIB-1 index compared to VHL-associated tumors (OR 2.35, 95% CI 1.05–5.27, *p* = 0.037), while the EoR did not confound this observation. **C** ROC curve illustrating the predictive value of MIB-1 labeling index for local tumor progression in VHL-associated sHB (AUC 0.74, 95% CI 0.49–0.98). **D** ROC curve for sporadic sHB, where MIB-1 index showed limited prognostic value (AUC 0.56, 95% CI 0.23–0.88)

Univariate analysis of the mean MIB-1 index between VHL-associated and sporadic sHB was performed using the independent *t*-test. In the entire patient cohort, the median MIB-1 labeling index was 3.0% (IQR: 1.0–10.0). Comparison of the MIB-1 index between VHL-associated and sporadic sHB revealed significantly lower values in the VHL-associated group (2.17% ± 1.58) compared to the sporadic group (3.02% ± 1.77) (*p* = 0.008). Figure 2A visualizes the comparison.

Further comparison of MIB-1 index values across various clinical and tumor-related variables showed no significant differences with respect to dichotomization by the median age (< 48 vs. > 48 years; *p* = 0.66), sex (*p* = 0.75), localization (cervical vs. non-cervical; *p* = 0.99), spinal compartment involvement (intramedullary vs. non-intramedullary; *p* = 0.58), multiplicity (solitary vs. multiple lesions; *p* = 0.655), number of involved segments (1 vs. ≥ 2; *p* = 0.99), presence of cysts (*p* = 0.88), presence of syrinx (*p* = 0.72), and presence of preoperative bleeding (*p* = 0.52).

A statistically significant difference was observed in relation to the EoR. Patients with incomplete resection had a significantly higher MIB-1 index compared to those with complete resection (3.93% vs. 2.39%, *p* = 0.025). For a detailed overview of these results, see Table 2.

**Table 2:** MIB-1 labeling indices among patient-, disease- and treatment-specific characteristics

Variable	MIB-1 index, mean (± SD)	*p*-value
Age		0.66
< 48	2.52 (± 1.75)	
> 48	2.67 (± 1.70)	
Female	2.53 (± 1.58)	0.75
Male	2.64 (± 1.87)	
Sporadic	3.02 (± 1.77)	*0.008*
VHL	2.17 (± 1.58)	
Intramedullary	2.66 (± 1.88)	0.58
Non-intramedullary	2.48 (± 1.46)	
Cervical	2.59 (± 1.78)	0.99
Non-cervical	2.58 (± 1.69)	
Multiple lesions	2.76 (± 2.31)	0.655
Solitary lesion	2.54 (± 1.54)	
Number of involved segments		0.99
1	2.56 (± 1.55)	
≥ 2	2.56 (± 2.16)	
Cyst		0.88
Present	2.61 (± 1.93)	
Absent	2.57 (± 1.59)	
Syrinx		0.72
Present	2.51 (± 1.62)	
Absent	2.63 (± 1.79)	
Preoperative bleeding		0.52
Present	3.00 (± 1.83)	
Absent	2.56 (± 1.72)	
Extent of resection		*0.025*
Complete resection	2.39 (± 1.53)	
Incomplete resection	3.93 (± 2.34)	

Italicized p-values indicate statistical significance at p < .05.

Multivariable binary logistic regression analysis of increased MIB-1 index (≥ 3%) considering EoR and tumor origin (VHL/sporadic) was performed. Sporadic status was an independent predictor for a MIB-1 index ≥ 3% (odds ratio (OR) = 2.35, 95% CI: 1.05–5.27, *p* = 0.037), while the EoR did not reach statistical significance (OR = 2.64, 95% CI: 0.67–10.45, *p* = 0.168). Figure 2B summarizes the results in a forest plot.

### Prognostic value of the MIB-1 index for local progression-free survival

ROC analysis revealed an area under the curve (AUC) of 0.74 (95% CI: 0.49–0.98) for the MIB-1 index regarding local PFS in VHL-associated sHBs (see Fig. 2C). Optimal cut-off value of MIB-1 index predicting local PFS in the VHL-associated sHBs was identified at ≥ 2%. The sensitivity and specificity at this threshold were 80.0% and 61.2%, respectively (Youden’s Index: 0.41).

**Fig. 3:**
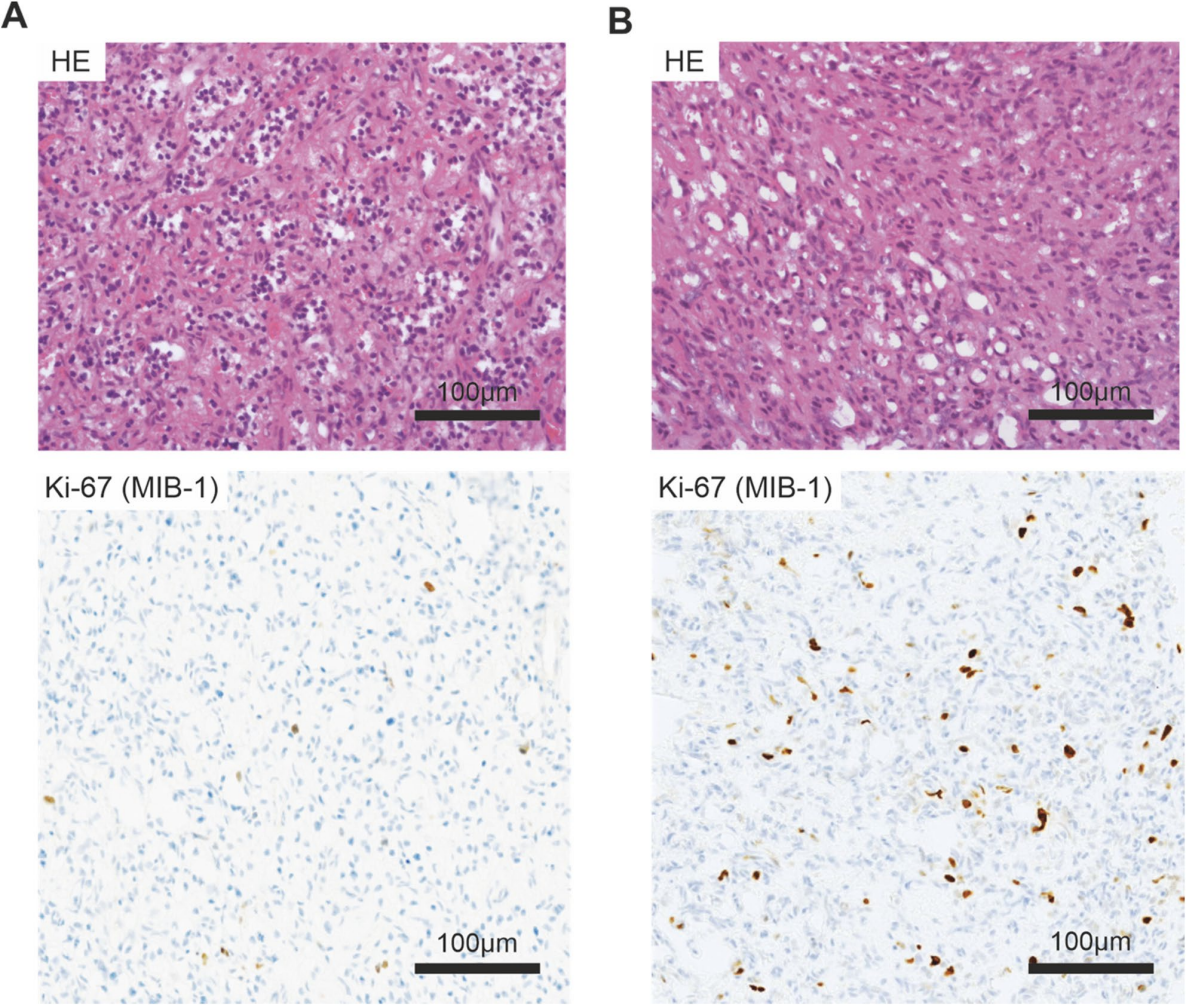
Representative microphotographs of tissue sections from spinal hemangioblastoma (sHB). Hematoxilin-Eosin staining (upper row) and Ki-67 (MIB-1) immunohistochemistry **A** VHL-associated sHB with low proliferative activity (1%) **B** sporadic sHB with high proliferative activity (5%)

In contrast, no relevant prognostic value was observed for the MIB-1 index in sporadic sHB (AUC = 0.56, 95% CI: 0.23–0.88, see Fig. 2D). Representative images demonstrating sHB cases with low (1%) versus high (5%) proliferative activity reflected by the MIB-1 index are shown in Fig. 3.

### Local progression-free survival

**Fig. 4:**
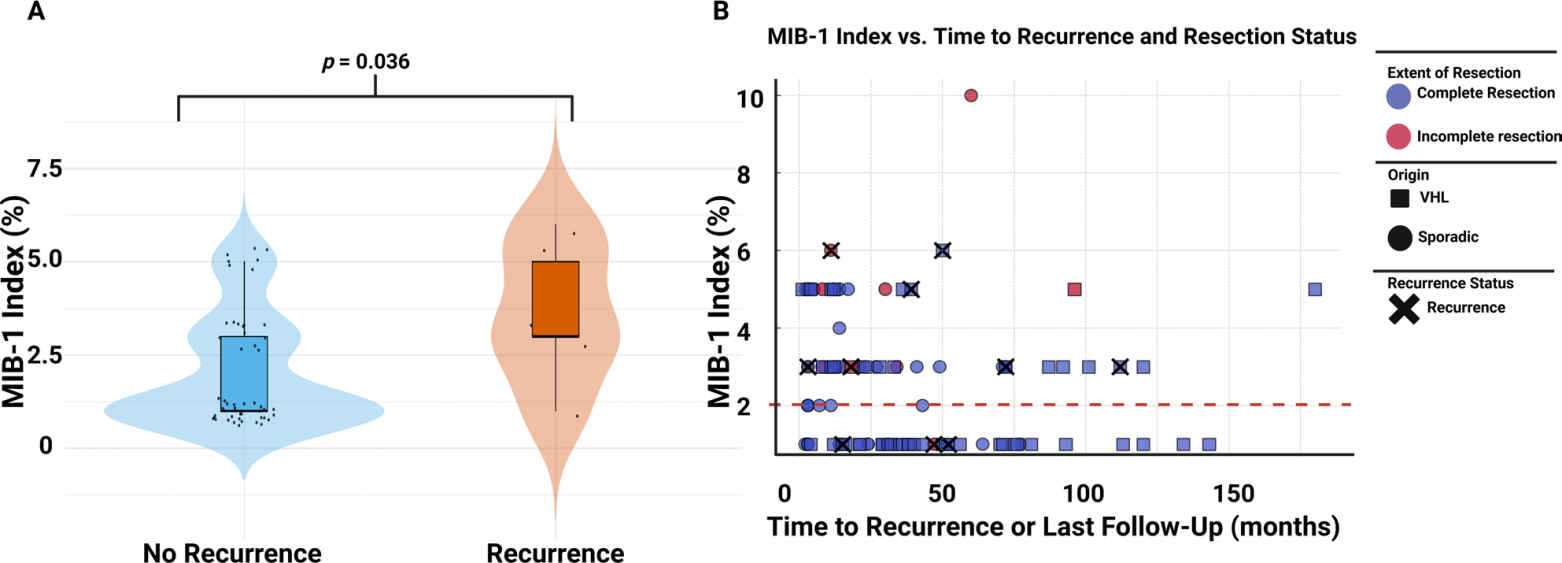
**A** Comparison of MIB-1 labeling index between patients with and without local tumor progression (Total: *n* = 97). Patients with local progression showed a significantly higher MIB-1 index (*p* = 0.036). **B** Bubble plot showing the relationship between MIB-1 index and time to local progression or last follow-up, with bubble colour indicating extent of resection (complete vs. incomplete) and line + size representing local progression status (Total: *n* = 97). Circles label those with a sporadic tumor, and squares those with a VHL-associated sHB. The black crosses indicate those cases who had a local recurrence. The red dashed line marks the MIB-1 index cut-off value of 2%

Furthermore, patients who experienced local tumor recurrence necessitating retreatment had significantly higher MIB-1 values than those without local progression (*p* = 0.036). These findings are further illustrated in Fig. 4, which visualizes the association between MIB-1 index and local tumor progression, highlighting significantly higher values in patients with shorter local PFS, as well as a clustering of elevated MIB-1 indices in early progression, particularly following incomplete resection.

In 97 (83.6%) patients both MIB-1 labeling indices and follow-up regarding local PFS was available. Median follow-up was 30.0 months (Range: 3.0–179.0 months). Local recurrence was observed in 10 cases (10/97; 10.3%). Kaplan–Meier analysis demonstrated a trend toward shorter PFS in patients with a MIB-1 index ≥ 2%, although this did not reach statistical significance in the overall cohort (*p* = 0.12). In the subgroup of sporadic sHB, no significant difference in PFS was observed between patients with MIB-1 < 2% and those with MIB-1 ≥ 2% (*p* = 0.87). The mean PFS in this subgroup was 59.6 months (95% CI: 40.3–78.8) for patients with MIB-1 < 2% and 66.9 months (95% CI: 58.4–75.3) for those with MIB-1 ≥ 2%.

**Fig. 5:**
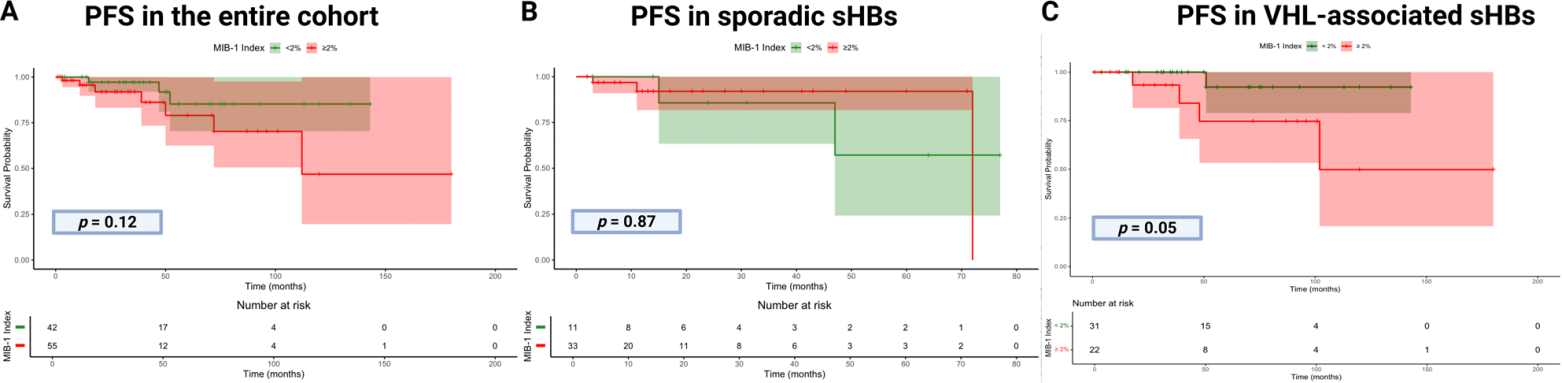
PFS according to MIB-1 labeling index stratified by subgroup. **A** In the entire cohort, higher MIB-1 index (≥ 2%) showed a trend toward shorter PFS without reaching statistical significance (*p* = 0.12). **B** In sporadic sHB, no significant difference in PFS was observed between high and low MIB-1 index groups (*p* = 0.87). The mean PFS was 59.6 months (95% CI: 40.3–78.8) for MIB-1 index < 2%, and 66.9 months (95% CI: 58.4–75.3) for MIB-1 ≥ 2%. **C** In VHL-associated sHB, a higher MIB-1 index (≥ 2%) was associated with significantly shorter PFS (*p* = 0.05). The mean PFS was 135.4 months (95% CI: 121.2–149.6) for MIB-1 < 2%, and 126.0 months (95% CI: 84.9–167.2) for MIB-1 index ≥ 2%

In contrast, among patients with VHL-associated sHB, a MIB-1 index ≥ 2% was significantly associated with shorter PFS (*p* = 0.05). The mean PFS was 135.4 months (95% CI: 121.2–149.6) in patients with MIB-1 < 2%, and 126.0 months (95% CI: 84.9–167.2) in those with MIB-1 ≥ 2%. Figure 5 summarizes the Kaplan–Meier analyses of local PFS.

In this subgroup of 97 patients with available follow-up, we additionally compared local PFS between sporadic and VHL-associated tumors to contextualize the prognostic relevance of MIB-1. Univariable Kaplan–Meier analysis showed a modest difference between groups (log-rank *p* = 0.04; Supplementary Fig. 1). However, when evaluating key clinical determinants of progression, extent of resection remained the dominant predictor, with incomplete resection showing a markedly higher risk of recurrence (log-rank *p* < 0.0001; Supplementary Fig. 2). Multivariable Cox regression confirmed this finding: incomplete resection was the only independent predictor of shorter PFS (HR 8.00, 95% CI 1.85–34.71), whereas tumor origin and MIB-1 ≥ 3% did not retain significance (Supplementary Fig. 3). These results indicate that the slight univariable PFS difference between sporadic and VHL-associated tumors is largely explained by differences in resection status rather than biological behavior. Importantly, this supports our main conclusion that elevated MIB-1 is prognostically meaningful only in VHL-associated sHB, where patients—especially those with incomplete resection—may warrant intensified postoperative monitoring or evaluation for adjuvant therapy.

## Discussion

The aim of this multicenter retrospective study was to evaluate the prognostic value of the MIB-1 index in patients with sHB, stratified by VHL-associated and sporadic cases. The results show that patients with VHL-associated sHB had significantly lower MIB-1 indices compared to those with sporadic sHB. Nevertheless, in VHL-associated sHB, a higher MIB-1 index with a cut-off value of ≥ 2% was significantly associated with an increased risk of progression and shorter local PFS. In contrast, no such association was observed in patients with sporadic sHB.

The MIB-1 labeling index, which reflects proliferative activity by measuring Ki-67 expression during the cell cycle, is an established prognostic marker for tumor growth, survival and local PFS in various CNS tumors [9, 15–17]. To date, published studies evaluating MIB-1 in hemangioblastomas have included at most 27 cases with immunohistochemical quantification [11]. The present study represents the largest cohort of sHB (*n* = 116) with systematically assessed MIB-1 indices and their association with local PFS. For example, a study by Miyagami et al., which analyzed 13 hemangioblastomas six of which were VHL-associated also demonstrated low MIB-1 indices, with a median value of 0.8% (range: 0.03–2.1%) [11]. However, in contrast to our findings, that study did not report a significant difference in MIB-1 values between VHL-associated and sporadic cases. Additional studies and case reports, using Ki-67 immunochemistry (often via the MIB-1 antibody), have consistently demonstrated low proliferative indices in both sporadic and VHL-associated hemangioblastomas [4, 18–21].These findings support the notion that hemangioblastomas, regardless of genetic background or anatomical location, typically exhibit low proliferative activity. Our study builds upon this evidence by identifying a prognostically relevant threshold (≥ 2%) for sHB specifically within the VHL subgroup, suggesting that even modest increases in proliferation may have clinical significance in genetically predisposed tumors.

The prognostic relevance of the MIB-1 index in sHB, particularly in VHL-associated cases, remains a subject of ongoing discussion. In our previous investigation including the entire cohort of 357 patients investigating patient-specific and treatment-specific parameters local PFS was primarily influenced by the EoR. Furthermore, local PFS did not significantly differ between VHL or sporadic cases [12]. Additional studies have also shown that functional outcome and local PFS are primarily influenced by the EoR [4–6]. Hence the present subgroup investigation reveals that the MIB-1 index seems to be a postoperative biomarker being of prognostic importance regarding local PFS in the subgroup of VHL-associated sHB.

The study by Hasselblatt et al. [22] investigated 88 hemangioblastomas and identified the cellular subtype as an independent risk factor for shorter local PFS, regardless of patient age, sex, or tumor localization. Tumors of the cellular subtype showed a higher MIB-1 index (median 4%) and shorter PFS compared to the reticular subtype. However, the study did not stratify cases by VHL status, and thus no conclusions could be drawn regarding the distribution of subtypes between VHL-associated and sporadic hemangioblastomas. In contrast, our study is the first to demonstrate a significant association between a MIB-1 index threshold of ≥ 2% and shorter PFS, specifically in VHL-associated sHB. No such association was observed in the sporadic subgroup. These findings provide novel evidence in a genetically defined patient population and suggest that even a moderate increase in proliferative activity, reflected by a MIB-1 index at or above this threshold, may be clinically relevant, even in the absence of a histologically defined cellular subtype.

The present study also has several limitations that should be considered. First, the retrospective design may introduce selection and information bias. Although the multicenter data acquisition increases the generalizability of the results, it also introduces variability in surgical techniques, pathological workup, and the assessment of the MIB-1 index. Given the retrospective multicenter design involving 13 participating centers across Europe and the United States, some variation in postoperative imaging intervals and follow-up scheduling was inevitable and reflected local institutional protocols and available VHL surveillance pathways. In patients with VHL-associated sHB, distinguishing between true local progression and the emergence of new primary tumors remains a clinical challenge, as these individuals are prone to multifocal disease [6]. Finally, although we identified a MIB-1 index threshold of ≥ 2% as being significantly associated with shorter PFS in VHL-associated sHB, external validation with equally sized cohorts is needed before this cut-off can be implemented as a reliable biomarker in clinical practice. This MIB-1 index cut-off might be a useful tool for a comprehensive consultation with patients and their relatives to determine more stringent follow-up intervals if an increased risk for a tumor progression. However, MIB-1 labeling indices in this investigation were determined over a long time period and by different neuropathologists in multiple institutions. Hence, observer bias might be present and necessitates future validation with digital image analysis [23]. Given the multicenter design, variability in MIB-1 staining across institutions is unavoidable and has been shown to affect interlaboratory consistency even under standardized conditions [24]. However, digital image analysis offers substantially improved reproducibility compared with manual counting, highlighting the need for future studies incorporating centralized or automated quantification [25].

In conclusion, our multicenter retrospective study demonstrated that the MIB-1 index in VHL-associated sHB is significantly lower than in sporadic cases. Nevertheless, we identified a MIB-1 index threshold at ≥ 2% in the VHL-associated group that seems to be associated with shorter local PFS. These findings suggest that even moderate increases in proliferative activity in VHL-associated sHB may have relevant clinical implications. In contrast, no prognostic relevance of the MIB-1 index was observed in sporadic cases. Our results underscore the need for further prospective validation studies to establish the MIB-1 index as a potential biomarker for individualized postoperative care in this genetically defined patient subgroup.

## Supplementary Information

Below is the link to the electronic supplementary material.

Additional Material 1

Additional Material 2

Additional Material 3

## Data Availability

The data sets generated and analyzed in the current study are available upon request from the corresponding author.

## Abbreviations

sHB: Spinal hemangioblastomas
VHL: Von Hippel-Lindau
PFS: Progression-free survival
AUC: Area under the curve
ROC: Receiver operating characteristics
QoL: Quality of life
CNS: Central nervous system
EoR: Extent of resection
IQR: Interquartile range
SD: Standard deviation
OR: Odds ratio
CI: Confidence interval
