# Supplementary material for: Prognostic relevance of MIB-1 labeling index in VHL-associated and sporadic spinal hemangioblastomas: a subgroup analysis from a multicentric study
Source: Acta Neuropathol Commun. 2025 Dec 11;14:18. doi: 10.1186/s40478-025-02202-w (PMC12801762; doi:10.1186/s40478-025-02202-w)
Supplement: Supplementary file 1 — Additional Material 1 [file 40478_2025_2202_MOESM1_ESM.pdf]

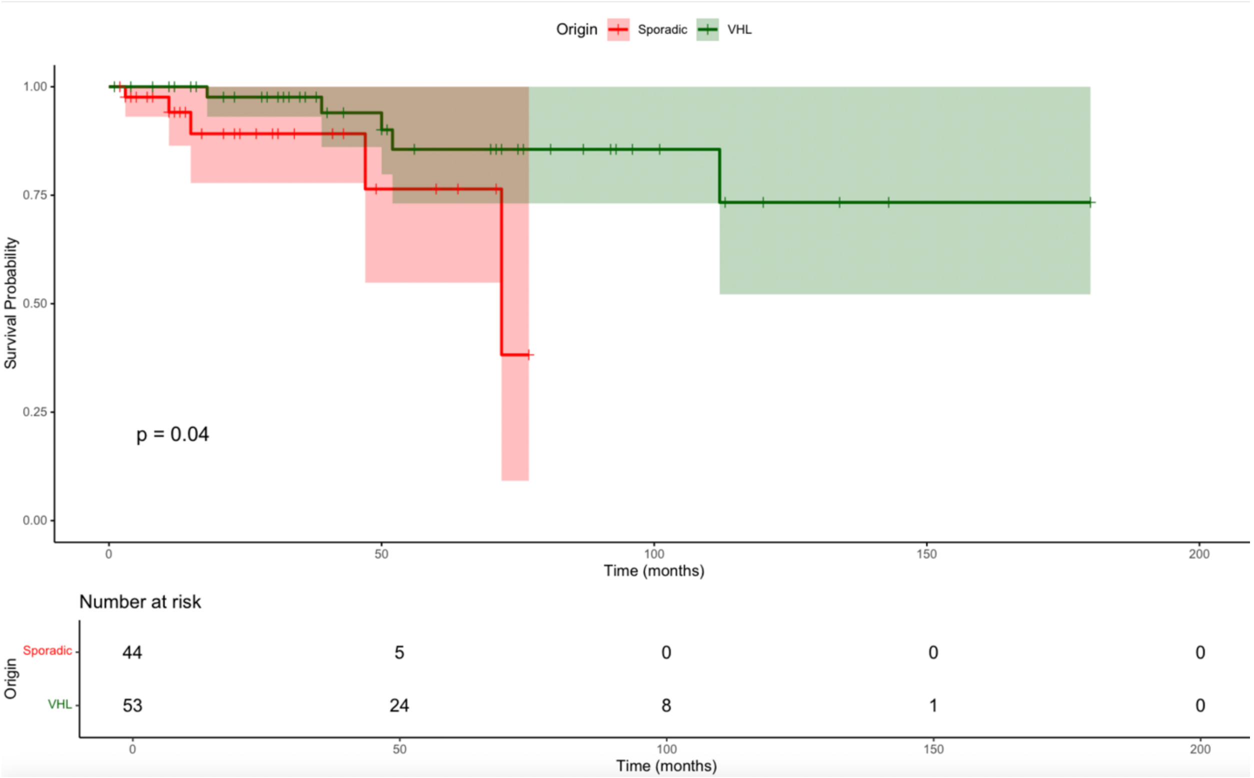

Supplementary figure 1 Kaplan–Meier curves of local progression-free survival by tumor origin: Kaplan–Meier curves illustrating local progression-free survival in patients with sporadic and VHL-associated spinal hemangioblastomas. The figure displays survival probabilities over time with shaded confidence bands and includes a number-at-risk table beneath
