# Supplementary material for: Prognostic relevance of MIB-1 labeling index in VHL-associated and sporadic spinal hemangioblastomas: a subgroup analysis from a multicentric study
Source: Acta Neuropathol Commun. 2025 Dec 11;14:18. doi: 10.1186/s40478-025-02202-w (PMC12801762; doi:10.1186/s40478-025-02202-w)
Supplement: Supplementary file 2 — Additional Material 2 [file 40478_2025_2202_MOESM2_ESM.pdf]

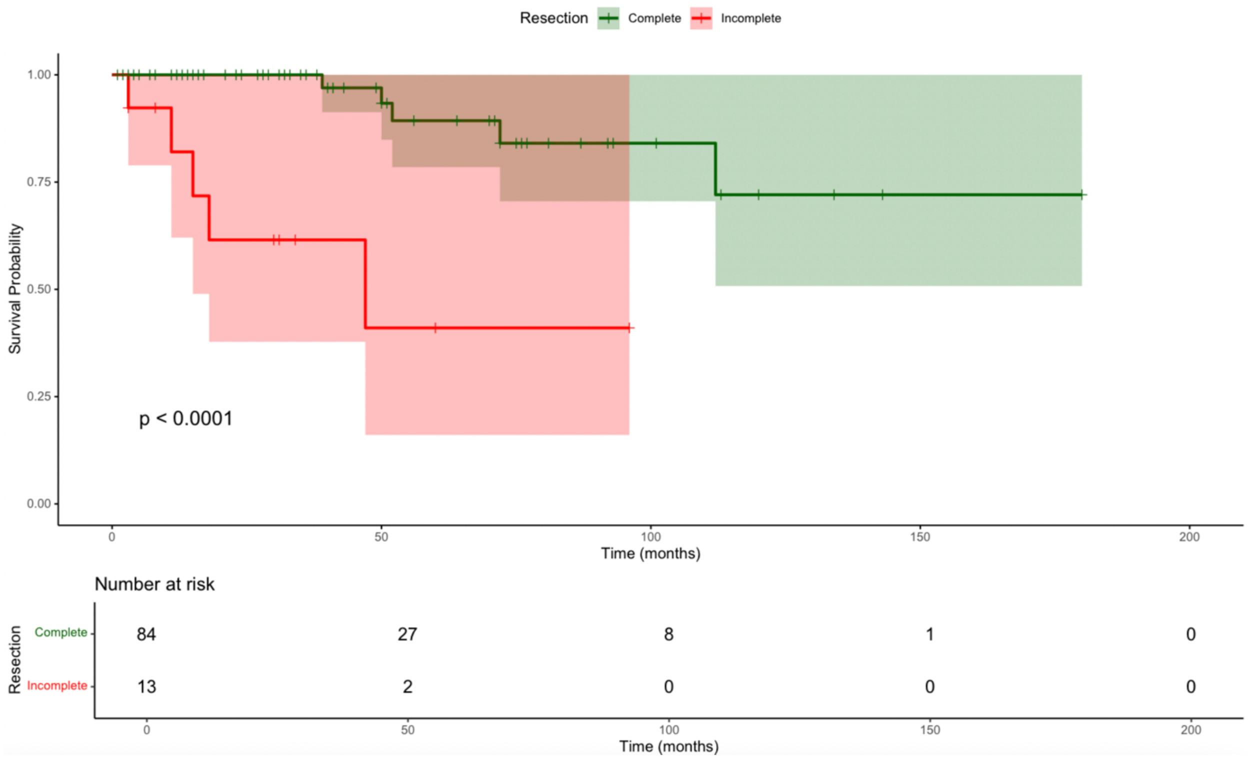

Supplementary Figure 2. Kaplan–Meier curves of local progression-free survival by extent of resection: Kaplan–Meier curves demonstrating local progression-free survival stratified by extent of resection (complete vs. incomplete). The plot presents time-to-event distributions with shaded confidence intervals and a corresponding number-at-risk table.
