# Supplementary material for: Prognostic relevance of MIB-1 labeling index in VHL-associated and sporadic spinal hemangioblastomas: a subgroup analysis from a multicentric study
Source: Acta Neuropathol Commun. 2025 Dec 11;14:18. doi: 10.1186/s40478-025-02202-w (PMC12801762; doi:10.1186/s40478-025-02202-w)
Supplement: Supplementary file 3 — Additional Material 3 [file 40478_2025_2202_MOESM3_ESM.pdf]

	Hazard ratio	95 % CI: Lower limit	95 % CI: Upper limit	*p*-value
MIB-1 ≥3%	1.76	0.43	7.26	0.44
Incomplete resection	8.00	1.85	34.71	0.005
Sporadic	1.87	0.42	8.36	0.41

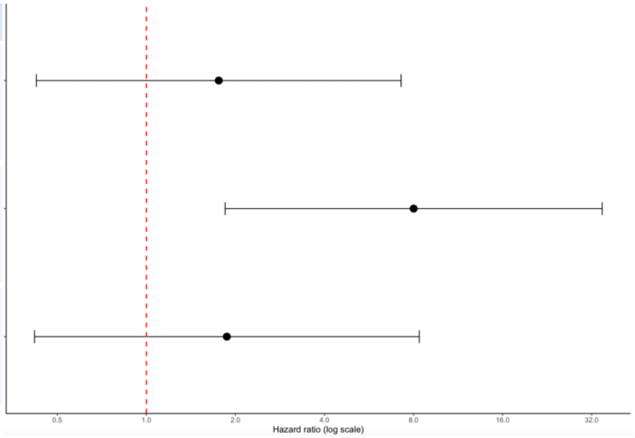

Supplementary Figure 3. Multivariable Cox regression model of predictors of local progression-free survival: Forest plot and accompanying table depicting a multivariable Cox regression model evaluating predictors of local progression-free survival. The figure displays hazard ratios with 95% confidence intervals on a logarithmic scale for each included variable.
